# The role of capecitabine-based neoadjuvant and adjuvant chemotherapy in early-stage triple-negative breast cancer: a systematic review and meta-analysis

**DOI:** 10.1186/s12885-021-07791-y

**Published:** 2021-01-19

**Authors:** Xingfa Huo, Jinming Li, Fuxing Zhao, Dengfeng Ren, Raees Ahmad, Xinyue Yuan, Feng Du, Jiuda Zhao

**Affiliations:** 1 Breast Disease Diagnosis and Treatment Center of Affiliated Hospital of Qinghai University & Affiliated Cancer Hospital of Qinghai University, Xining, 810000 China; 2 Peking University Cancer Hospital and Institute, Beijing, 100142 China

**Keywords:** Triple-negative breast cancer, Capecitabine, Neo/adjuvant chemotherapy, Survival

## Abstract

**Background:**

The role of capecitabine in neoadjuvant and adjuvant chemotherapy for early-stage triple-negative breast cancer (TNBC) is highly controversial. Our meta-analysis was designed to further elucidate the effects of capecitabine on survival in early-stage TNBC patients and its safety.

**Methods:**

PubMed, Embase, and papers presented at several main conferences were searched up to December 19, 2019, to investigate capecitabine-based versus capecitabine-free neoadjuvant and adjuvant chemotherapy in TNBC patients. Heterogeneity was assessed using *I*^2^ test, combined with hazard ratios (HRs) and odds ratios (ORs) with 95% confidence intervals (CI) computed for disease-free survival (DFS), overall survival (OS), and over grade 3 adverse events (AEs).

**Results:**

A total of 9 randomized clinical trials and 3842 TNBC patients were included. Overall, the combined capecitabine regimens in neoadjuvant and adjuvant chemotherapy showed significantly improved DFS (HR = 0.75; 95% CI, 0.65–0.86; *P* < 0.001) and OS (HR = 0.63; 95% CI, 0.53–0.77; *P* < 0.001). In subgroup analysis, there were improvements in DFS in the groups with addition of capecitabine (HR = 0.64; 95% CI, 0.53–0.78; *P* < 0.001), adjuvant chemotherapy (HR = 0.73; 95% CI, 0.63–0.85; *P* < 0.001), and lymph node positivity (HR = 0.62; 95% CI, 0.44–0.86; *P* = 0.005). Capecitabine regimens were related to higher risks of diarrhea (OR = 2.88, 95% CI 2.23–3.74, *P* < 0.001), stomatitis (OR = 2.01, 95% CI 1.53–2.64, *P* < 0.001) and hand–foot syndrome (OR = 8.67, 95% CI 6.70–11.22, *P* < 0.001).

**Conclusion:**

This meta-analysis showed that neoadjuvant and adjuvant chemotherapy combined with capecitabine significantly improved both DFS and OS in early-stage TNBC patients with tolerable AEs. There were benefits to DFS in the groups with the addition of capecitabine, adjuvant chemotherapy, and lymph node positivity.

**Supplementary Information:**

The online version contains supplementary material available at 10.1186/s12885-021-07791-y.

## Background

“Triple-negative” [negative for estrogen receptor (ER−), progesterone receptor (PR−), and human epidermal growth factor receptor 2 (HER-2)] breast cancers (TNBCs) account for approximately 15 to 20% of all breast cancers and have the characteristics of heterogeneity, aggressiveness, and poor prognosis [1–3]. Although programmed cell death 1 (PD1), programmed cell death ligand 1 (PD-L1) inhibitor, and poly (ADP-ribose) polymerase (PARP) inhibitor have been shown to be effective in the neoadjuvant phase [4–7], chemotherapy is still the backbone of treatment by neoadjuvant and adjuvant therapy [8]. National Comprehensive Cancer Network Guidelines and the St. Gallen International Expert Consensus recommended standard regimens containing anthracycline and paclitaxel for early-stage breast cancer patients [9, 10]. However, even if the chemotherapy regimen is effective, the 10-year risk of relapse of early TNBC is still up to 20–40% [11–13]. Therefore, it is crucial to explore new adjuvant and neoadjuvant chemotherapy drugs and regimens, improve the treatment efficacy, and translate any progress into survival benefits.

Capecitabine is one of the most widely studied drugs in the neoadjuvant and postoperative adjuvant setting of TNBC. Recently, several randomized clinical trials (RCTs) have evaluated the clinical value of combined treatment with capecitabine for TNBC, but the outcomes of these studies were still controversial [14–22]. Seven meta-analyses summarizing the role of capecitabine in breast cancer adjuvant and neoadjuvant chemotherapy have been performed, six of which included all subtypes of patients, not just TNBC patients [13, 23–28]. Some meta-analyses estimated disease-free survival (DFS) of TNBC in a subgroup, and only three reported overall survival (OS) [13, 26, 28], but the DFS and OS of TNBC results were controversial. Although a meta-analysis was recently performed to investigate the role of capecitabine in early-stage TNBC [13], it included only seven studies; it appeared that most of the participants in the research by Zhang et al. were hormone receptor- and/or HER-2-positive, while the meta-analysis dealt with all patients having TNBC. In addition, none of the meta-analyses mentioned above included the latest data of two studies (CBCSG-010 and CIBOMA) that particularly focused only on TNBC, and also did not distinguish neoadjuvant chemotherapy from adjuvant chemotherapy as it appeared that patients could not benefit from the addition of capecitabine to neoadjuvant chemotherapy [29]. Our meta-analysis enlarges the sample size and refines the subgroup analysis possible to make the conclusion more robust.

To further clarify the role of capecitabine in TNBC, in the present study, we conducted a meta-analysis to investigate the effect of capecitabine as neoadjuvant and adjuvant chemotherapy on survival in TNBC patients.

## Methods

This analysis was based on the Preferred Reporting Items for Systematic Reviews and Meta-Analyses (PRISMA) guidelines [30].

### Search criteria

Randomized controlled trials (RCTs) were identified using a computerized search of the databases PubMed and Embase (up to December 2019), using the MeSH terms “breast cancer,” “breast neoplasms,” “triple-negative breast cancer,” “triple-negative breast neoplasms,” “capecitabine,” “Xeloda,” “neoadjuvant chemotherapy,” and “adjuvant chemotherapy.” The search was limited to articles written in English. We also searched the proceedings of the last 10 years of annual meetings at the American Society of Clinical Oncology, European Society for Medical Oncology, and San Antonio Breast Cancer Symposium, including relevant unpublished studies. We used the following criteria: (i) phase II or III RCTs; (ii) TNBC patients who received neoadjuvant or adjuvant chemotherapy including capecitabine in the experimental arm and chemotherapy without capecitabine in the control arm; and (iii) studies reporting DFS and/or OS risk ratios. We excluded those with no reported hazard ratios (HRs) of 95% confidence intervals (CI) for DFS or recurrence-free survival (RFS) and OS in TNBC, as well as non-RCT, meta-analysis, and retrospective studies.

### Data extraction

Two investigators (XH and FZ) independently extracted data of the RCTs by a search strategy, and then proceeded to aggregate the results; divergence was resolved by consensus. The following information was extracted: trial name, authors’ names, update year, trial design, trial phase, number of TNBC patients, median follow-up, hormone receptor and human epidermal growth factor receptor 2 (HER-2) statuses, and chemotherapy regimens. The HRs and 95% CI of the efficacy measures for DFS and OS were extracted where available, and grade 3–5 drug-related adverse events (AEs) were also extracted. For studies with multiple reported outcomes, the latest reported data were used. The Cochrane Collaboration’s tool for assessing risk of bias was used to assess the quality and potential bias of studies (Table S1).

### Statistical analysis

The combined HRs of each of DFS and OS were weighted and combined by the generic inverse variance method [31]. Pooled estimates of odds ratios (ORs) were computed for grade 3–5 drug-related AEs. Subgroup analysis was conducted according to (i) adding and replacing capecitabine in chemotherapy, (ii) neoadjuvant and adjuvant chemotherapy, or (iii) lymph node positivity and negativity. Heterogeneity was assessed using *I*^2^ test [32]. When *P* < 0.10 or *I*^2^ > 50%, the random effects model was used, and when *P* > 0.10 or *I*^2^ < 50%, the fixed effects model was used [33]. The possibility of publication bias was estimated by observing a funnel plot (Figure S1). *P* < 0.05 was considered significant. All statistical analyses were performed using Stata 12.0 software.

## Results

### Characteristics of the studies

A total of 508 records were retrieved based on the search terms; after excluding 499 irrelevant records, nine potentially eligible RCTs were considered [14–22], one of which was a conference abstract [21] (Figure S2). However, the data for the FinXX trial are based on RFS, not DFS. Because the definition of DFS in the other trials resembles that of RFS in the FinXX trial, we combined the RFS of FinXX with the DFS of the other trials to perform a joint analysis. A total of 3842 TNBC patients were included in this meta-analysis. In two of the trials, the entire cohort of patients had TNBC [19, 21], while in the other seven trials TNBC patients were one of the subgroups. The main research features are shown in Table 1.

**Table 1:** Main characteristics of the studies included in the meta-analysis

Study	Author	Update year	Trial Phase	Capecitabine arm	Control arm	*N* (Capecitabine/ Control)	TNBC, *N* (X/Control)	Median follow-up (month)	Design	Setting
FinXX Trial	Joensuu	2017	III	DX + CEX	D + CEF	751/744	93/109	123.6	Addition	Adjuvant
GEICAM/2003–10	Martín	2015	III	ED-X	EC-D	715/669	95/71	79.2	Replace	Adjuvant
GAIN	V. Mo	2017	III	EC-PX	EPC	1511/1512	213/208	74	Replace	Adjuvant
US ncology 01062	O’Shaughnessy	2015	III	AC-DX	AC-D	1307/1304	396/384	60	Addition	Adjuvant
CREATE-X	Masuda	2017	III	X	None	443/444	139/147	43.2	Addition	Adjuvant
CIBOMA 2004/01	Lluch	2019	III	ED-X	EC-D	448/428	448/428	87.6	Addition	Neo/Adjuvant
Gepar TRIO	Minckwitz	2013	III	DAC-NX	DAC-DAC	301/321	NA	62	Replace	Neoadjuvant
CBCSG-010	Li	2019	III	DX-XEC	D-FEC	297/288	297/288	67	Addition	Adjuvant
CALGB49907	Muss	2019	III	X	CMF/AC	307/326	76/78	136.8	Replace	Adjuvant

*X* capecitabine, *C* cyclophosphamide, *M* methotrexate, *F* 5-fluorouracil, *A* doxorubicin, *E* epirubicin, *D* docetaxel, *BEV* bevacizumab, *P* paclitaxel, *N* nab-paclitaxe, *NA* not available, *N* number

### DFS and OS

**Fig. 1:**
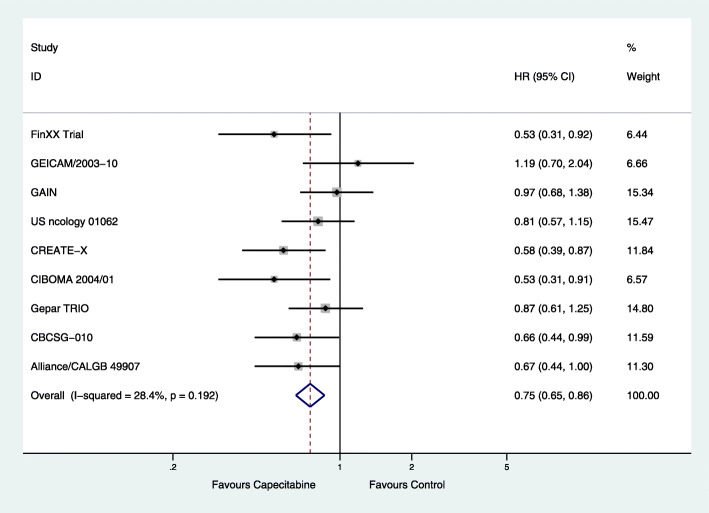
Effects of combined capecitabine regimens in neoadjuvant and adjuvant chemotherapy on disease-free survival (DFS)

**Fig. 2:**
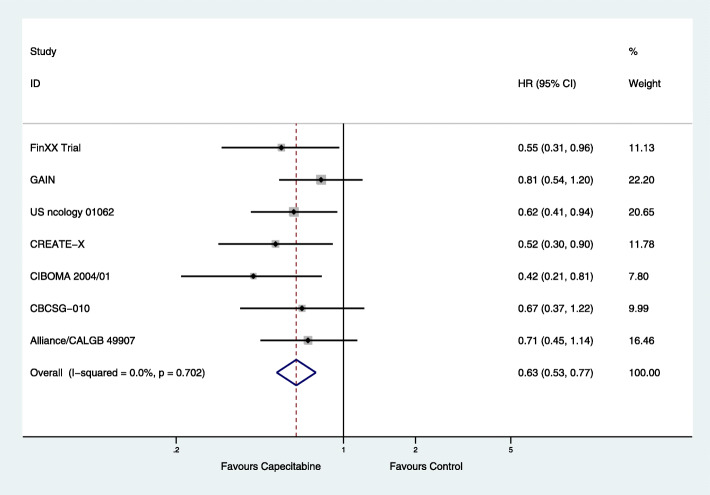
Effects of combined capecitabine regimens in neoadjuvant and adjuvant chemotherapy on overall survival (OS)

The HRs of nine RCTs for both DFS and OS were pooled. From the available data, there were 1757 cases of involving the addition or replacement of capecitabine in the capecitabine group and 1425 cases of chemotherapy without capecitabine. The combined capecitabine regimens in neoadjuvant and adjuvant chemotherapy were associated with significantly improved DFS (HR = 0.75; 95% CI, 0.65–0.86; *P* < 0.001) with low heterogeneity (*P* = 0.192, *I*^2^ = 28.4%) (Fig. 1). They also significantly improved OS (HR = 0.63; 95% CI, 0.53–0.77; *P* < 0.001) with low heterogeneity (*P* = 0.702, *I*^2^ = 0) (Fig. 2).

### DFS subgroup analysis

#### Addition and replacement of capecitabine

**Fig. 3:**
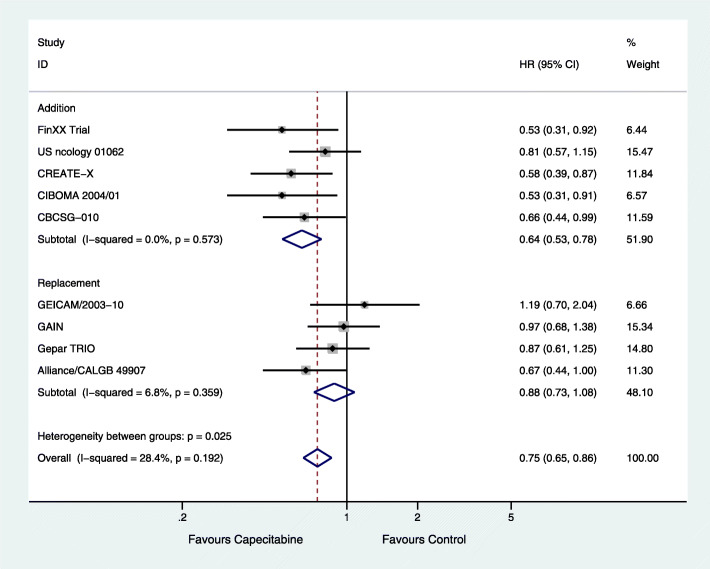
Effects of the addition or replacement of capecitabine on subgroup analysis of disease-free survival (DFS)

There were five [14, 17–19, 21] and four [15, 16, 20, 22] studies involving the addition or replacement of capecitabine to neoadjuvant or adjuvant chemotherapy, respectively. From the available data of TNBC, there were 1373 capecitabine combined and 1356 capecitabine-free cases in the group with the addition of capecitabine, and 384 capecitabine combined and 357 capecitabine-free cases in the group with the replacement of capecitabine. DFS was significantly improved in the group with the addition of capecitabine (HR = 0.64; 95% CI, 0.53–0.78; *P* < 0.001) with high heterogeneity (*P* = 0.573, *I*^2^ = 0), but not in the group with the replacement of capecitabine (HR = 0.88; 95% CI, 0.73–1.08; *P* = 0.225) with low heterogeneity (*P* = 0.359, *I*^2^ = 6.8%) (Fig. 3).

#### Adjuvant and neoadjuvant chemotherapy

**Fig. 4:**
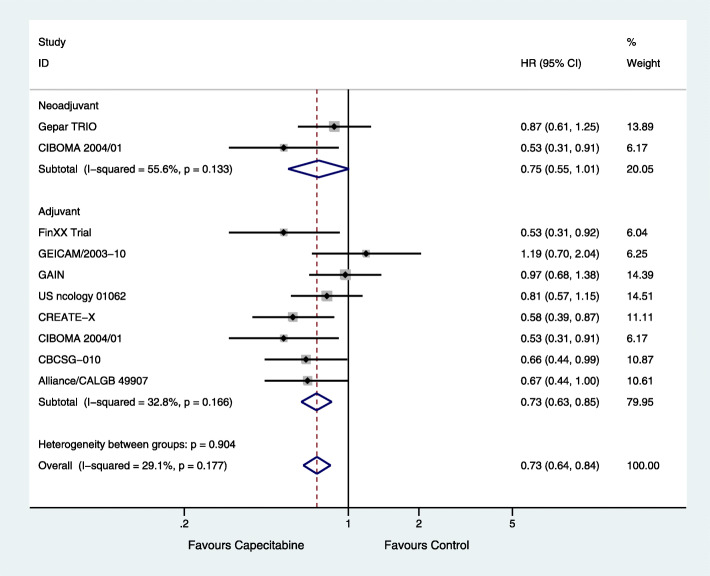
Effects of using capecitabine as neoadjuvant or adjuvant chemotherapy on subgroup analysis of disease-free survival (DFS)

There were seven [14–19, 21, 22] and one [20] studies that used capecitabine as adjuvant and neoadjuvant chemotherapy, respectively, and one study that used capecitabine as both neoadjuvant and adjuvant chemotherapy [19]. From the available data of TNBC, there were 1757 cases of adjuvant chemotherapy with capecitabine and 1713 cases of adjuvant chemotherapy without capecitabine, along with 448 cases of neoadjuvant chemotherapy with capecitabine and 428 cases of neoadjuvant chemotherapy without capecitabine. DFS was significantly improved upon using capecitabine adjuvant chemotherapy (HR = 0.73; 95% CI, 0.63–0.85; *P* < 0.001) with low heterogeneity (*P* = 0.166, *I*^2^ = 32.8%), but not upon using capecitabine neoadjuvant chemotherapy (HR = 0.75; 95% CI, 0.55–1.01; *P* = 0.056) with low heterogeneity (*P* = 0.133, *I*^2^ = 55.6%) (Fig. 4).

#### Lymph node status

**Fig. 5:**
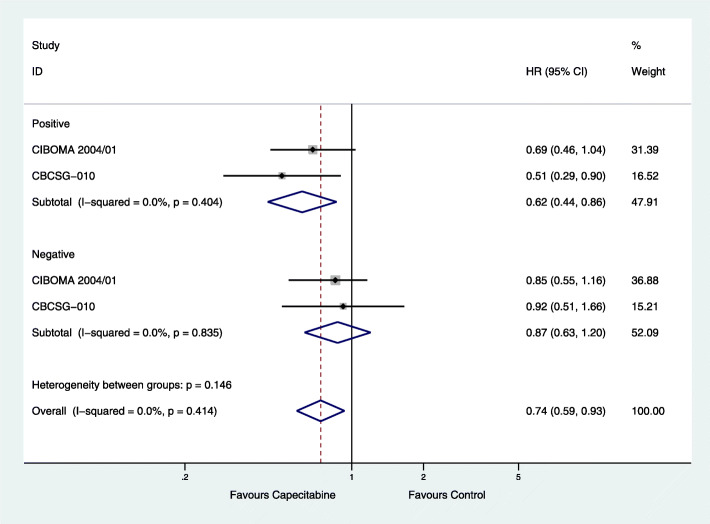
Effects of combined capecitabine regimens in neoadjuvant and adjuvant chemotherapy in different lymph node statuses on subgroup analysis of disease-free survival (DFS)

Two studies (CIBOMA 2004/01, CBCSG-010) [19, 21] reported that there is a relationship between lymph node status of TNBC patients and neoadjuvant or adjuvant chemotherapy including capecitabine. There were 295 lymph node-positive cases and 440 lymph node-negative cases in the capecitabine group, along with 285 lymph node-positive cases and 427 lymph node-negative cases in the capecitabine-free group. DFS was significantly improved in lymph node-positive patients (HR = 0.62; 95% CI, 0.44–0.86; *P* = 0.005) with low heterogeneity (*I*^2^ = 0%, *P* = 0.404), but not in lymph node-negative patients (HR = 0.87; 95% CI, 0.63–1.20; *P* = 0.403) with low heterogeneity (*I*^2^ = 0%, *P* = 0.835) (Fig. 5).

#### Grade 3–5 adverse event analysis

**Fig. 6:**
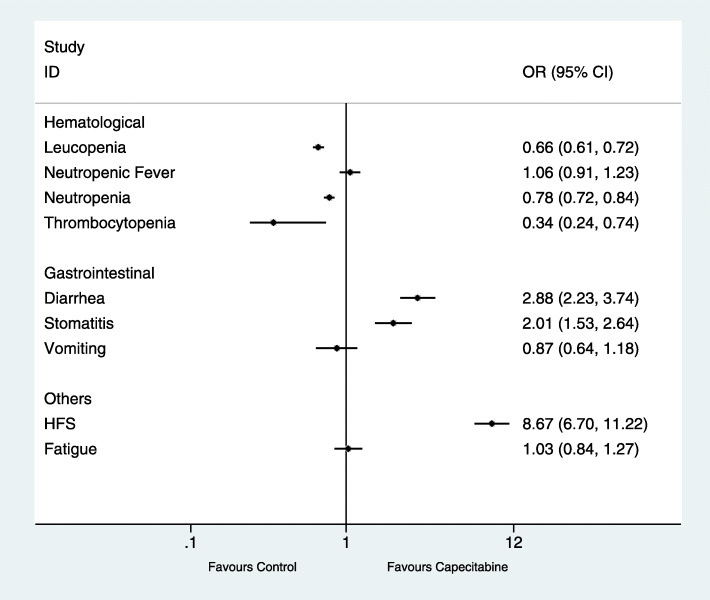
An overview of the safety profile for grade 3–5 adverse events (AEs) in capecitabine and capecitabine-free chemotherapy groups

Considering that the influences of adverse events related to hormone receptor and HER-2 statuses were small, we used the overall population’s AE data to perform this meta-analysis. In total, 4682 of 37,675 (12.43%) patients were in the capecitabine group and 5029 of 37,276 (13.49%) were in the capecitabine-free group. There were four grade 3–5 hematological AEs, three grade 3–5 gastrointestinal AEs, and two grade 3–5 other AEs. Figure 6 shows an overview of the safety profile for grade 3–5 AEs in the capecitabine and capecitabine-free chemotherapy groups.

#### Hematological AEs

In six RCTs, the rates of grade 3–4 leucopenia were reported [14, 16–20]. In total, 1225 of 4761 (25.73%) patients in the capecitabine group developed grade 3–4 leucopenia after neoadjuvant or adjuvant treatment and 1859 of 4753 (39.11%) did so in the capecitabine-free group. The capecitabine arms showed a lower risk of leucopenia (OR = 0.66, 95% CI 0.61–0.72, *P* < 0.001) with significant heterogeneity (*P* < 0.001, *I*^2^ = 92.7%) (Fig. 6).

In seven RCTs, the rates of grade 3–4 neutropenia were reported [14–18, 20, 21]. Overall, 1681 of 5352 (31.41%) patients in the capecitabine group developed grade 3–4 neutropenia after neoadjuvant or adjuvant treatment and 2100 of 5282 (39.76%) did so in the capecitabine-free group. The capecitabine arms showed a lower risk of neutropenia (OR = 0.78, 95% CI 0.72–0.84, *P* < 0.001) with significant heterogeneity (*P* < 0.001, *I*^2^ = 94.2%) (Fig. 6).

In six RCTs, the rates of grade 3–4 thrombocytopenia were reported [14, 16, 18–21]. In total, 40 of 3751 (1.07%) patients in the capecitabine group developed grade 3–4 thrombocytopenia after neoadjuvant or adjuvant treatment and 141 of 3737 (3.77%) did so in the capecitabine-free group. The capecitabine arms showed a lower risk of thrombocytopenia (OR = 0.34, 95% CI 0.24–0.47, *P* < 0.001) with significant heterogeneity (*P* < 0.001, *I*^2^ = 82.8%) (Fig. 6).

#### Gastrointestinal AEs

In seven RCTs, the rates of grade 3–5 diarrhea were reported [14–19, 21]. Overall, 235 of 5472 (4.29%) patients in the capecitabine group developed grade 3–5 diarrhea after neoadjuvant or adjuvant treatment and 81 of 5389 (1.50%) did so in the capecitabine-free group. The capecitabine arms showed a higher risk of diarrhea (OR = 2.88, 95% CI 2.23–3.74, *P* < 0.001) with significant heterogeneity (*P* = 0.02, *I*^2^ = 62.8%) (Fig. 6).

In five RCTs, the rates of grade 3–5 stomatitis were reported [14, 17, 18, 20, 21]. In total, 177 of 3099 (5.71%) patients in the capecitabine group developed grade 3–5 stomatitis after neoadjuvant or adjuvant treatment and 83 of 3101 (2.68%) did so in the capecitabine-free group. The capecitabine arms showed a higher risk of stomatitis (OR = 2.01, 95% CI 1.53–2.64, *P* < 0.001) with significant heterogeneity (*P* = 0.025, *I*^2^ = 67.9%) (Fig. 6).

#### Others AEs

In six RCTs, the rates of grade 3 hand–foot syndrome (HFS) were reported [14, 15, 17–19, 21]. Overall, 589 of 3961 (14.87%) patients in the capecitabine group developed grade 3 HFS after neoadjuvant or adjuvant treatment and 66 of 3877 (1.70%) did so in the capecitabine-free group. The capecitabine arms showed a higher risk of HFS (OR = 8.67, 95% CI 6.70–11.22, *P* < 0.001) with significant heterogeneity (*P* < 0.001, *I*^2^ = 80.8%) (Fig. 6).

## Discussion

To the best of our knowledge, this is the most comprehensive meta-analysis based on the most recent studies which evaluates the efficacy and safety of neoadjuvant and adjuvant chemotherapy regimens combined with capecitabine for TNBC. With nine studies and a total of 3842 TNBC patients in this meta-analysis, the neoadjuvant and adjuvant chemotherapy combined with capecitabine for TNBC patients not only improved DFS, but also improved OS. In subgroup analysis, benefits to DFS were identified in the groups with the addition of capecitabine, adjuvant chemotherapy, and lymph node positivity, but not in those with the replacement of capecitabine, neoadjuvant chemotherapy, and lymph node negativity. Capecitabine regimens were related to higher risks of grade 3–5 AEs for diarrhea, stomatitis, and HFS, but lower risks of leucopenia, neutropenia, and thrombocytopenia, while there were no significant differences in the AEs neutropenic fever, fatigue, and vomiting compared with the case for capecitabine-free regimens.

Capecitabine has been used for treating advanced breast cancer and is efficient after advancement in an anthracycline- or taxane-based setting [34–36]. Previous studies also showed that capecitabine may have specific benefits for TNBC patients [37, 38]. Considering the efficacy of capecitabine for treatment in a metastatic setting and TNBC, speculation has been reported about its potential role in early TNBC and several large clinical trials on this issue have been carried out. However, these studies reported conflicting results and few studies focused on TNBC. The overview of seven meta-analyses can be found in Table S2. A meta-analysis included all subtypes of breast cancer patients, and TNBC patients were analyzed in subgroup analysis only [23]. But in our meta-analysis, we included only patients with TNBC and subgroup analysis included the addition/replacement of capecitabine, adjuvant/neoadjuvant chemotherapy, and lymph node status, we also increased the analysis of AEs of stomatitis. Recently, a meta-analysis from Li et al. [13] reported that TNBC patients might benefit from the addition of capecitabine in the adjuvant settings, however, this meta-analysis not only included TNBC patients, but included hormone receptor- and/or HER-2-positive patients. In our article, we analyzed TNBC patients, and two latest study results (GEICAM and CBCSG-010). Although several meta-analyses based on limited studies and patients summarized the role of capecitabine in neoadjuvant and adjuvant chemotherapy for breast cancer [13, 26, 28], the results regarding DFS and OS in TNBC were still controversial.

More recently, three studies (CALGB 49907, GEICAM/2003-11_CIBOMA/2004–01, and CBCSG-010) provided updated results, two of which only included TNBC patients treated with capecitabine adjuvant or neoadjuvant chemotherapy. Thus, it is necessary to reevaluate the efficacy of capecitabine in neoadjuvant and adjuvant chemotherapy. Our results revealed the significant extension of DFS and OS by capecitabine neoadjuvant and adjuvant chemotherapy in early-stage TNBC patients. For patients with positivity for hormone receptor or HER-2, the effects of adjuvant treatment can be intensified by inhibiting ovarian function and prolonging endocrine therapy [39–43] or by other anti-HER-2 drugs including pertuzumab, neratinib, and TDM1 [44–46]. Based on our results, capecitabine might become an intensive treatment protocol for patients with TNBC.

Overall, five studies (FinXX Trial, US Oncology 01062, CREATE-X, CIBOMA 2004/01, CBCSG-010) and four studies (GEICAM/2003–10, GAIN, Gepar TRIO, CALGB49907) have been performed on the addition or replacement of capecitabine in neoadjuvant or adjuvant chemotherapy, respectively. The E1199, ABC, and S0221 trials suggested that the inclusion of anthracyclines in addition to taxanes and alkylator chemotherapy is effective for early-stage TNBC patients [47–49]. The chemotherapy regimens were all based on anthracyclines and/or taxanes in the group with the addition of capecitabine in our meta-analysis, so DFS can be further significantly improved. However, the replacement of capecitabine with anthracycline and/or taxane will compromise the survival of patients.

This meta-analysis also revealed that there was a survival benefit of using capecitabine only in adjuvant chemotherapy, but not in a neoadjuvant setting. Two meta-analyses [29, 50] showed no significant improvement in the rate of pathologic complete response (pCR) in neoadjuvant treatment combined with capecitabine. Achieving pCR after neoadjuvant treatment can significantly improve DFS in TNBC patients [51–53]. The negative relationship between capecitabine and pCR in a neoadjuvant setting might account for capecitabine not improving the DFS of TNBC patients. Nevertheless, there was a significant improvement in the rate of DFS in the group with adjuvant chemotherapy combined with capecitabine, which may be related to the long-term effects of capecitabine on dormant tumor cells, activating anti-cancer immunity or antiangiogenic activity [54–56].

Only two studies (CIBOMA 2004/01, CBCSG-010) reported the relationship between lymph node status of TNBC patients and neoadjuvant or adjuvant chemotherapy including capecitabine. Our meta-analysis showed that survival benefits were only conferred in lymph node-positive patients. Lymph node metastasis is one of the risk factors for TNBC patients [19]. Patients who did not reach pCR in the CREATE-X trial [18] and received capecitabine could benefit from capecitabine as intensive treatment. Thus, capecitabine might be more efficacious for high-risk TNBC patients. Similarly, capecitabine only improved the survival of lymph node-positive patients, but not lymph node-negative ones.

We also evaluated the AEs associated with capecitabine as neoadjuvant and adjuvant chemotherapy. Our meta-analysis showed that capecitabine-based neoadjuvant and adjuvant chemotherapy increased the risks of diarrhea, stomatitis, and HFS, but reduced the risks of grade 3–5 hematological AEs such as leucopenia, neutropenia, and thrombocytopenia. Physicians should be aware of the possibility of several capecitabine-related AEs in clinical practice, such as HFS, diarrhea, and stomatitis, which might lead to the discontinuation of treatment. The neoadjuvant and adjuvant capecitabine combined chemotherapy was negatively related to the risk of grade 3–5 hematological toxicities, which may be associated with the replacement of capecitabine monotherapy in some capecitabine groups. Nevertheless, the toxicity of capecitabine was acceptable and easily manageable in the patients overall.

Our meta-analysis has some limitations. First, this is a meta-analysis based on the literature but not on individual patient data. Thus, biased findings might have been obtained regarding the treatment efficacy and toxicity. Second, the studies included in this meta-analysis have several potential differences, such as the definition of TNBC and the chemotherapy regimens. In some studies, the rate of cases of TNBC as defined by ER/PR expression was less than 10% [14], while in other studies it was less than 1% [19]. The most used chemotherapy regimens were based on anthracyclines, taxanes, and cyclophosphamide, but there were still some differences among the studies, such as the dose of chemotherapeutics, and one study also used antiangiogenic drugs. This heterogeneity should be taken into account when interpreting our findings. Third, the results of one study were only provided in conference abstract form, but not as a full report. Some information was incomplete, such as patient age, and dose and duration of chemotherapy. Fourth, the types of AEs reported in the included studies differed, and some AEs were not reported in several studies. Considering that the influences of AEs related to hormone receptor and HER-2 status were small, we used the overall population’s AE data to perform this meta-analysis, but the comprehensive effect of AEs may be biased compared with the actual results.

## Conclusion

In conclusion, our meta-analysis showed that neoadjuvant and adjuvant chemotherapy combined with capecitabine significantly improved both DFS and OS in early-stage TNBC patients. DFS was significantly improved in the groups with the addition of capecitabine, adjuvant chemotherapy, and lymph node positivity, but not in those with the replacement of capecitabine, neoadjuvant chemotherapy, or lymph node negativity. Capecitabine regimens were related to higher risks of grade 3–5 AEs, namely, diarrhea, stomatitis, and HFS, but negatively correlated with the risks of leucopenia, neutropenia, and thrombocytopenia.

## Supplementary Information

**Additional file 1: Table S1**. Assessment of bias of randomized controlled trials.

**Additional file 2: Table S2**. The main findings/conclusions of previous meta-analyses.

**Additional file 3: Figure S1**. Funnel plot for disease-free survival (DFS).

**Additional file 4: Figure S2**. Search strings and flow charts for filtering and research selection.

## Data Availability

Not applicable.

## Abbreviations

TNBC: Triple-negative breast cancer
HRs: Hazard ratios
ORs: Odds ratios
CI: Confidence intervals
DFS: Disease-free survival
OS: Overall survival
AEs: Adverse events
RCTs: Randomized clinical trials
ER: Estrogen receptor
PR: Progesterone receptor
HER-2: Human epidermal growth factor receptor 2
PD1: Programmed cell death 1
PD-L1: Programmed cell death ligand 1
PARP: Poly (ADP-ribose) polymerase
RFS: Recurrence-free survival
PRISMA: Preferred Reporting Items for Systematic Reviews and Meta-Analyses
HFS: Hand–foot syndrome
pCR: Pathologic complete response

## References

[1] Metzger-Filho O, Tutt A, de Azambuja E, Saini KS, Viale G, Loi S (2012). Dissecting the heterogeneity of triple-negative breast cancer. J Clin Oncol.

[2] Lehmann BD, Bauer JA, Chen X, Sanders ME, Chakravarthy AB, Shyr Y (2011). Identification of human triple-negative breast cancer subtypes and preclinical models for selection of targeted therapies. J Clin Invest.

[3] Fitzpatrick A, Tutt A (2019). Controversial issues in the neoadjuvant treatment of triple-negative breast cancer. Ther Adv Med Oncol.

[4] Fasching PA, Jackisch C, Rhiem K, Schneeweiss A, Klare P, Hanusch C, et al. A randomized phase II trial to assess the efficacy of paclitaxel and olaparib in comparison to paclitaxel/carboplatin followed by epirubicin/cyclophosphamide as neoadjuvant chemotherapy in patients (pts) with HER2-negative early breast cancer (BC) and homologous recombination deficiency (HRD). J Clin Oncol. 2019:506.

[5] ART B, S L: Triple-negative breast cancer: recent treatment advances. F1000Research 2019, 8. 10.12688/f1000research.18888.1 PMC6681627 31448088

[6] Schmid P, Cortés J, Dent R, Pusztai L, McArthur H L, Kuemmel S, et al. KEYNOTE-522: Phase III study of pembrolizumab (pembro) + chemotherapy (chemo) vs placebo (pbo)+ chemo as neoadjuvant treatment, followed by pembro vs pbo as adjuvant treatment for early triple-negative breast cancer (TNBC). Ann Oncol. 2019;30(suppl 5). mdz394.003.

[7] Eikesdal HP, Yndestad S, Blix ES, Lundgren S, Vagstad G, Espelid H (2019). 184PD Neoadjuvant olaparib monotherapy in primary triple negative breast cancer. Ann Oncol.

[8] Sonnenblick A, Piccart M (2015). Adjuvant systemic therapy in breast cancer: quo vadis?. Ann Oncol.

[9] Waks AG, Winer EP (2019). Breast cancer treatment: a review. JAMA..

[10] Curigliano G, Burstein HJ, Winer PE, Gnant M, Dubsky P, Loibl S (2017). De-escalating and escalating treatments for early-stage breast cancer: the St. Gallen international expert consensus conference on the primary therapy of early breast cancer 2017. Ann Oncol.

[11] Peto R, Davies C, Early Breast Cancer Trialists' Collaborative Group (EBCTCG) (2012). Comparisons between different polychemotherapy regimens for early breast cancer: meta-analyses of long-term outcome among 100,000 women in 123 randomised trials. Lancet.

[12] Ali AM, Ansari J, El-Aziz N, Abozeed WN, Warith A, Alsaleh K (2017). Triple-negative breast cancer: a tale of two decades. Anti Cancer Agents Med Chem.

[13] Y L, Y Z, F M, Y L, X Z, S S, et al. (2020). Adjuvant addition of capecitabine to early-stage triple-negative breast cancer patients receiving standard chemotherapy: a meta-analysis. Breast Cancer Res Treat.

[14] Joensuu H, Kellokumpu-Lehtinen PL, Huovinen R, Jukkola-Vuorinen A, Tanner M, Kokko R (2017). Adjuvant capecitabine in combination with docetaxel, epirubicin, and cyclophosphamide for early breast cancer: the randomized clinical FinXX trial. JAMA Oncol.

[15] Martín M, Ruiz Simón A, Ruiz Borrego M, Ribelles N, Rodríguez-Lescure Á, Muñoz-Mateu M (2015). Epirubicin plus cyclophosphamide followed by docetaxel versus epirubicin plus docetaxel followed by capecitabine as adjuvant therapy for node-positive early breast cancer: results from the GEICAM/2003-10 study. J Clin Oncol.

[16] von Minckwitz G, Möbus V, Schneeweiss A, Huober J, Thomssen C, Untch M (2013). German adjuvant intergroup node-positive study: a phase III trial to compare oral ibandronate versus observation in patients with high-risk early breast cancer. J Clin Oncol.

[17] O'Shaughnessy J, Koeppen H, Xiao Y, Lackner MR, Paul D, Stokoe C (2015). Patients with slowly proliferative early breast cancer have low five-year recurrence rates in a phase III adjuvant trial of capecitabine. Clin Cancer Res.

[18] Masuda N, Lee SJ, Ohtani S (2017). Adjuvant capecitabine for breast cancer after preoperative chemotherapy. N Engl J Med.

[19] Lluch A, Barrios CH, Torrecillas L, Ruiz-Borrego M, Bines J, Segalla J (2020). Phase III trial of adjuvant Capecitabine after standard neo−/adjuvant chemotherapy in patients with early triple-negative breast Cancer (GEICAM/2003-11_CIBOMA/2004-01). J Clin Oncol.

[20] von Minckwitz G, Blohmer JU, Costa SD, Denkert C, Eidtmann H, Eiermann W (2013). Response-guided neoadjuvant chemotherapy for breast cancer. J Clin Oncol.

[21] Li J, Yu K, Pang D, Wang C, Jiang J, Yang S (2020). Adjuvant Capecitabine with Docetaxel and cyclophosphamide plus Epirubicin for triple-negative breast Cancer (CBCSG010): an open-label, randomized, multicenter. Phase III Trial JCO.

[22] Muss HB, Polley MC, Berry DA, Liu H, Cirrincione CT, Theodoulou M (2019). Randomized trial of standard adjuvant chemotherapy regimens versus capecitabine in older women with early breast cancer: 10-year update of the CALGB 49907 trial. J Clin Oncol.

[23] Natori A, Ethier JL, Amir E, Cescon DW (2017). Capecitabine in early breast cancer: a meta-analysis of randomised controlled trials. Eur J Cancer.

[24] Chen G, Guo Z, Liu M, Yao G, Dong J, Guo J (2017). Clinical value of capecitabine-based combination adjuvant chemotherapy in early breast cancer: a meta-analysis of randomized controlled trials. Oncol Res.

[25] Zhang ZC, Xu QN, Lin SL, Li XY (2016). Capecitabine in combination with standard (neo) adjuvant regimens in early breast cancer: survival outcome from a meta-analysis of randomized controlled trials. PLoS One.

[26] Xu D, Chen X, Li X, Mao Z, Tang W, Zhang W (2019). Addition of Capecitabine in breast Cancer first-line chemotherapy improves survival of breast Cancer patients. J Cancer.

[27] Jiang Y, Yin W, Zhou L, Yan T, Zhou Q, Du Y (2012). First efficacy results of capecitabine with anthracycline-and taxane-based adjuvant therapy in high-risk early breast cancer: a meta-analysis. PLoS One.

[28] van Mackelenbergh M, Seither F, Möbus V, O'Shaugnessy J, Martin M, Joenssuu H (2020). Abstract GS1–07: Effects of capecitabine as part of neo−/adjuvant chemotherapy. A meta-analysis of individual patient data from 12 randomized trials including 15,457 patients. Cancer Res.

[29] Li Y, Yang D, Chen P, Yin X, Sun J, Li H (2019). Efficacy and safety of neoadjuvant chemotherapy regimens for triple-negative breast cancer: a network meta-analysis. Aging (Albany NY).

[30] Liberati A, Altman DG, Tetzlaff J, Mulrow C, Gøtzsche PC, Ioannidis J (2009). The PRISMA statement for reporting systematic reviews and meta-analyses of studies that evaluate health care interventions: explanation and elaboration. PLoS Med.

[31] DerSimonian R, Laird N (1986). Meta-analysis in clinical trials. Controlled clinical trials. Control Clin Trials.

[32] Mantel N, Haenszel W (1959). Statistical aspects of the analysis of data from retrospective studies of disease. J Natl Cancer Inst.

[33] Zintzaras E, Ioannidis JP (2005). Heterogeneity testing in meta-analysis of genome searches. Genet Epidemiol.

[34] Blum JL, Jones SE, Buzdar AU, LoRusso PM, Kuter I, Vogel C, et al. Multicenter phase II study of capecitabine in paclitaxel-refractory metastatic breast cancer. J Clin Oncol. 1999;17(2):485–5. 10.1200/JCO.1999.17.2.485 10080589

[35] O'Shaughnessy J, Miles D, Vukelja S, Moiseyenko V, Ayoub JP, Cervantes G (2002). Superior survival with capecitabine plus docetaxel combination therapy in anthracycline-pretreated patients with advanced breast cancer: phase III trial results. J Clin Oncol.

[36] Varshavsky-Yanovsky AN, Goldstein LJ (2020). Role of Capecitabine in early breast Cancer. J Clin Oncol.

[37] Kotsori AA, Dolly S, Sheri A, Parton M, Shaunak N, Ashley S (2010). Is capecitabine efficacious in triple negative metastatic breast cancer?. Oncology..

[38] Chacón RD, Costanzo MV (2010). Triple-negative breast cancer. Breast Cancer Res.

[39] Goldvaser H, Barnes TA, Šeruga B, Cescon DW, Ocaña A, Ribnikar D (2018). Toxicity of extended adjuvant therapy with aromatase inhibitors in early breast cancer: a systematic review and meta-analysis. JNCI: Journal of the National Cancer Institute.

[40] Bartlett J, Sgroi DC, Treuner K, Zhang Y, Ahmed I, Piper T (2019). Breast Cancer index and prediction of benefit from extended endocrine therapy in breast cancer patients treated in the adjuvant Tamoxifen—to offer more?(aTTom) trial. Ann Oncol.

[41] Gnant M, Mlineritsch B, Stoeger H, Luschin-Ebengreuth G, Knauer M, Moik M (2014). Zoledronic acid combined with adjuvant endocrine therapy of tamoxifen versus anastrozol plus ovarian function suppression in premenopausal early breast cancer: final analysis of the Austrian breast and colorectal Cancer study group trial 12. Ann Oncol.

[42] Davies C, Pan H, Godwin J, Gray R, Arriagada R, Raina V (2013). Long-term effects of continuing adjuvant tamoxifen to 10 years versus stopping at 5 years after diagnosis of oestrogen receptor-positive breast cancer: ATLAS, a randomised trial. Lancet.

[43] Goss PE, Ingle JN, Pritchard KI, Robert NJ, Muss H, Gralow J (2016). Extending aromatase-inhibitor adjuvant therapy to 10 years. N Engl J Med.

[44] Dong R, Ji J, Liu H, He X (2019). The evolving role of trastuzumab emtansine (T-DM1) in HER2-positive breast cancer with brain metastases. Crit Rev Oncol Hematol..

[45] Xuhong JC, Qi XW, Zhang Y, Jiang J (2019). Mechanism, safety and efficacy of three tyrosine kinase inhibitors lapatinib, neratinib and pyrotinib in HER2-positive breast cancer. Am J Cancer Res.

[46] Kalimutho M, Parsons K, Mittal D, López JA, Srihari S, Khanna KK (2015). Targeted therapies for triple-negative breast cancer: combating a stubborn disease. Trends Pharmacol Sci.

[47] Blum JL, Flynn PJ, Yothers G, Asmar L, Geyer CE, Jr Jacobs SA (2017). Anthracyclines in early breast Cancer: the ABC trials-USOR 06-090, NSABP B-46-I/USOR 07132, and NSABP B-49 (NRG oncology). J Clin Oncol.

[48] Sparano JA, Zhao F, Martino S, Ligibel JA, Perez EA, Saphner T (2015). Long-term follow-up of the E1199 phase III trial evaluating the role of taxane and schedule in operable breast cancer. J Clin Oncol.

[49] Budd GT, Barlow WE, Moore HC, Hobday TJ, Stewart JA, Isaacs C (2015). SWOG S0221: a phase III trial comparing chemotherapy schedules in high-risk early-stage breast cancer. J Clin Oncol.

[50] Li Q, Jiang Y, Wei W, Yang H, Liu J (2013). Clinical efficacy of including capecitabine in neoadjuvant chemotherapy for breast cancer: a systematic review and meta-analysis of randomized controlled trials. PLoS One.

[51] von Minckwitz G, Untch M, Blohmer JU, Costa SD, Eidtmann H, Fasching PA (2012). Definition and impact of pathologic complete response on prognosis after neoadjuvant chemotherapy in various intrinsic breast cancer subtypes. J Clin Oncol.

[52] Cortazar P, Zhang L, Untch M, Mehta K, Costantino JP, Wolmark N (2014). Pathological complete response and long-term clinical benefit in breast cancer: the CTNeoBC pooled analysis. Lancet.

[53] Spring LM, Fell G, Arfe A, Sharma C, Greenup RA, Reynolds KL (2020). Pathological complete response after neoadjuvant chemotherapy and impact on breast cancer recurrence and survival: a comprehensive meta-analysis. Clin Cancer Res.

[54] Gnoni A, Silvestris N, Licchetta A, Santini D, Scartozzi M, Ria R (2015). Metronomic chemotherapy from rationale to clinical studies: a dream or reality?. Crit Rev Oncol Hematol.

[55] Klement G, Baruchel S, Rak J, Man S, Clark K, Hicklin DJ (2000). Continuous low-dose therapy with vinblastine and VEGF receptor-2 antibody induces sustained tumor regression without overt toxicity. J Clin Invest.

[56] Zitvogel L, Apetoh L, Ghiringhelli F, Kroemer G (2008). Immunological aspects of cancer chemotherapy. Nat Rev Immunol.

