# Supplementary material for: The role of capecitabine-based neoadjuvant and adjuvant chemotherapy in early-stage triple-negative breast cancer: a systematic review and meta-analysis
Source: BMC Cancer. 2021 Jan 19;21:78. doi: 10.1186/s12885-021-07791-y (PMC7816481; doi:10.1186/s12885-021-07791-y)
Supplement: Supplementary file 1 — Additional file 1: Table S1. Assessment of bias of randomized controlled trials. [file 12885_2021_7791_MOESM1_ESM.docx]

Table S1. Assessment of bias of randomized controlled trials.

| **Study** | **Year** | **Randomization** | **Allocation concealment** | **Blinding** | **Incomplete outcome data** | **Selective reporting** | **Other sources of bias** |
| --- | --- | --- | --- | --- | --- | --- | --- |
| FinXX Trial | 2017 | Low | High | Low | Low | Low | Low |
| GEICAM/2003-10 | 2015 | Low | High | Low | Low | Low | Low |
| GAIN | 2017 | Low | High | Low | Low | Low | Low |
| US ncology 01062 | 2015 | Low | High | Low | Low | Unknown | Low |
| CREATE-X | 2017 | Low | Low | Low | Low | Low | Low |
| CIBOMA 2004/01 | 2019 | Low | Low | Low | Unknown | Unknown | Low |
| Gepar TRIO | 2013 | Low | High | High | Low | Unknown | Low |
| CBCSG-010 | 2019 | Low | Low | Unknown | Unknown | Low | Low |
| CALGB 49907 | 2019 | Low | High | Low | Unknown | Low | Low |
