# Supplementary material for: The role of capecitabine-based neoadjuvant and adjuvant chemotherapy in early-stage triple-negative breast cancer: a systematic review and meta-analysis
Source: BMC Cancer. 2021 Jan 19;21:78. doi: 10.1186/s12885-021-07791-y (PMC7816481; doi:10.1186/s12885-021-07791-y)
Supplement: Supplementary file 2 — Additional file 2: Table S2. The main findings/conclusions of previous meta-analyses. [file 12885_2021_7791_MOESM2_ESM.docx]

**Table S2 The main findings/conclusions of previous meta-analyses.**

| Title | Author | Year | Number of the studies | Inclusion criteria | Results | Main conclusion |
| --- | --- | --- | --- | --- | --- | --- |
| Adjuvant addition of capecitabine to early‑stage triple‑negative breast cancer patients receiving standard chemotherapy: a meta‑analysis | Yan Li | 2019 | 7 | 1. Early-stage breast cancer patients (e.g., TNBC); 2. The RCT studies was included; 3. One arm: standard (neo)adjuvant chemotherapy;   The other arm: addition of capecitabine to the adjuvant settings besides standard chemotherapy;   1. Standard chemotherapy was defined as anthracycline-/taxane-based regimens; 2. The RCT reported HRs with 95% CIs of DFS or RFS and OS. | DFS (HR = 0.77, 95% CI 0.66 –0.90, *p* = 0.001);  OS (HR = 0.69, 95% CI 0.56 –0.85, *p* = 0.001). | Adjuvant addition of capecitabine to early-stage TNBC patients receiving standard chemotherapy showed significant DFS and OS improvement. |
| Capecitabine in early breast cancer: A meta-analysis of randomised controlled trials | Akina Natori | 2017 | 8 | 1. RCTs that compared standard chemotherapy with or without capecitabine in the neoadjuvant or adjuvant setting. 2. Standard chemotherapy was defined as cyclophosphamide, methotrexate, and 5-fluorouracil, anthracycline-based regimens or anthracycline/taxane combinations. 3. The studies reporting hazard ratios (HRs) for OS or DFS. | DFS (HR = 0.99, 95% CI 0.80 – 1.22, *P* = 0.93);  OS (HR = 0.90, 95% CI 0.73 – 1.12, *P* = 0.36). | Adding capecitabine to standard chemotherapy appears to improve DFS and OS in TNBC, but increases adverse events in keeping with its known toxicity profile. |
| Clinical Value of Capecitabine-Based Combination Adjuvant Chemotherapy in Early Breast Cancer: A Meta-Analysis of Randomized Controlled Trials | Guanling Chen | 2017 | 8 | 1. Patients with operable, nonmetastatic breast cancer; 2. RCTs that compared capecitabine-based regimens with capecitabine-free regimens in a combination adjuvant chemotherapy setting. | DFS (HR =0.96, 95% CI = 0.89 –1.05, *P* = 0.38);  OS (HR = 0.91, 95% CI = 0.82 – 1.00, *P* = 0.06). | Capecitabine-based combination adjuvant chemotherapy might provide some breast cancer-specific survival benefit in early breast cancer. However, the absolute survival gain is small, as no improvement was observed in DFS, OS, or relapse. |
| Capecitabine in Combination with Standard (Neo)Adjuvant Regimens in Early Breast Cancer: Survival Outcome from a Meta-Analysis of Randomized Controlled Trials | Ze-Chun Zhang | 2016 | 7 | 1. Prospective phase II or III RCTs; 2. Capecitabine in the (neo)adjuvant setting of breast cancer; 3. RCTs reporting HRs with 95% CI of DFS and OS. | DFS (HR = 0.93; 95% CI, 0.85 –1.02; *P* = 0.12);  OS (HR = 0.85; 95% CI, 0.75 –0.96; *P* = 0.008). | Combining capecitabine with standard (neo)adjuvant regimens in early breast cancer demonstrated a significantly superior OS, and indicated DFS improvement in some subtypes with high risk of recurrence. |
| Addition of Capecitabine in Breast Cancer First-line  Chemotherapy Improves Survival of Breast Cancer  Patients | Di Xu | 2019 | 10 | 1. Studies with breast cancer patients; 2. Patients receive capecitabine-based combination with first-line (neo)adjuvant treatment; 3. Randomized, open-label, phase III clinical trial; 4. Studies that evaluated the efficacy of capecitabine in the neoadjuvant treatment; 5. The reported data of outcomes DFS or OS were sufficient to calculate; 6. Reporting HR with a 95% CI. | TNBC patients:  DFS (HR = 0.77, 95% CI: 0.65 –0.92; *P* = 0.004);  OS (HR = 0.65, 95% CI: 0.51 –0.81; *P* < 0.001). | Capecitabine combined with first-line chemotherapy in the treatment of breast cancer is an effective and safe treatment option and is worthy of clinical application to improve survival of breast cancer patients. |
| First Efficacy Results of Capecitabine with Anthracyclineand Taxane-Based Adjuvant Therapy in High-Risk Early Breast Cancer: A Meta-Analysis | Yiwei Jiang | 2012 | 2 | Randomized, open-label, phase III trials in early breast cancer. | DFS (HR = 0.83, 95% CI: 0.71 –0.98, *P* = 0.027);  OS (HR = 0.71, 95% CI: 0.57 –0.88, *P* = 0.002). | Due to the synergistic effect of taxane and capecitabine, taxane-anthracycline-capecitabine regimen may effectively improve the efficacy in the adjuvant setting and may be a novel generation of adjuvant chemotherapy regimen. |
| Abstract GS1-07: Effects of capecitabine as part of neo-/adjuvant chemotherapy. A meta-analysis of individual patient data from 12 randomized trials including 15,457 patients | Marion van Mackelenbergh | 2020 | 12 | 1. Use of capecitabine in EBC as adjuvant or neoadjuvant therapy; 2. Randomized; 3. N>100 patients; 4. Recruitment completed and outcomes available. | The addition of capecitabine did not alter DFS significantly compared to treatment without capecitabine (HR = 0.952; 95% CI 0.895 – 1.012; *P* = 0.115). | Capecitabine did not alter DFS in this meta-analysis of 15,457 patients with early breast cancer from 12 prospective randomized trials, but as addition to systemic treatment DFS was improved. |

DFS: disease free survival OS: overall survival HRs: hazard ratios TNBC: triple-negative breast cancer CI: confidence interval
