# Supplementary figures and images for: The role of capecitabine-based neoadjuvant and adjuvant chemotherapy in early-stage triple-negative breast cancer: a systematic review and meta-analysis
Source: BMC Cancer. 2021 Jan 19;21:78. doi: 10.1186/s12885-021-07791-y (PMC7816481; doi:10.1186/s12885-021-07791-y)

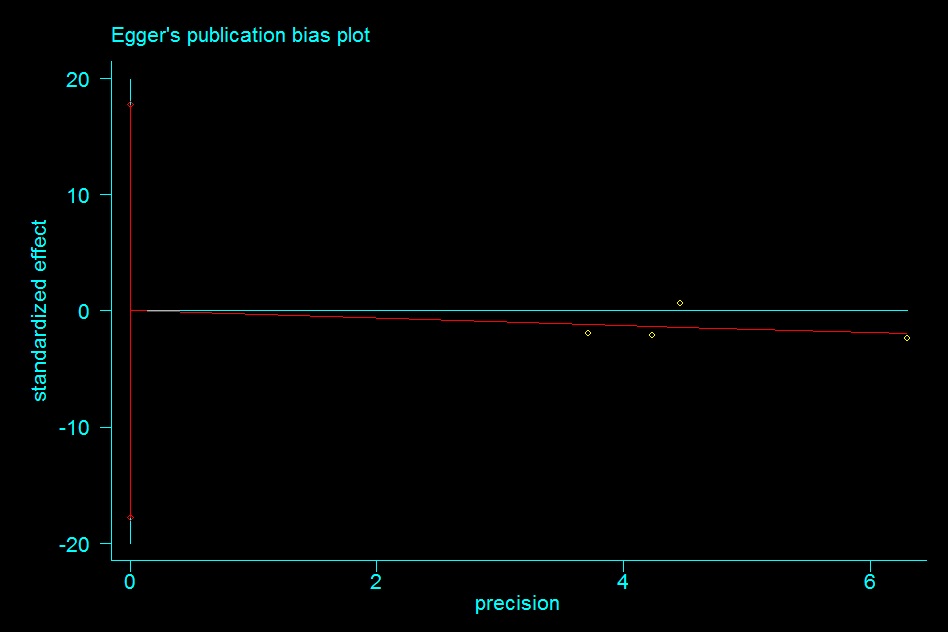

Supplement: Supplementary file 3 — Additional file 3: Figure S1. Funnel plot for disease-free survival (DFS). [file 12885_2021_7791_MOESM3_ESM.jpg]
