# Supplementary material for: The role of capecitabine-based neoadjuvant and adjuvant chemotherapy in early-stage triple-negative breast cancer: a systematic review and meta-analysis
Source: BMC Cancer. 2021 Jan 19;21:78. doi: 10.1186/s12885-021-07791-y (PMC7816481; doi:10.1186/s12885-021-07791-y)
Supplement: Supplementary file 4 — Additional file 4: Figure S2. Search strings and flow charts for filtering and research selection. [file 12885_2021_7791_MOESM4_ESM.docx]

Additional records identified through meeting websites: ASCO; ESMO; SABCS

(n=5)

**Identification**

Records identified through database searching (PubMed, EMBASE) (n=503)

Titles and abstracts screened (n=205)

Records after duplicates removed (n=303)

**Screening**

Records excluded (n=98)

**Eligibility**

Full-text articles assessed for eligibility (n=107)

Full-text articles excluded:

- Retrospective studies (n=23)
- No triple-negative subtype information (n=16)
- Duplicate data (n=5)
- Case report (n=7)
- Reviews (n=25)
- Metastasis disease (n=16)
- Date availiable (n=3)

Studies included in qualitative synthesis (n=12)

**Included**

Studies included in quantitative synthesis (meta-analysis) (n=9)

Figure S2. Search strings and flow charts for filtering and research selection.
